# Gray matter volume and functional connectivity identify asymptomatic MAPT variant carriers with signs of longitudinal disease progression

**DOI:** 10.1002/alz70856_105877

**Published:** 2026-01-10

**Authors:** Youjin Jung, Taru M. Flagan, Liwen Zhang, Maria Luisa Mandelli, Virginia E. Sturm, Jennifer S. Yokoyama, Maria Luisa Gorno Tempini, Brad F. Boeve, Adam L Boxer, Leah K. Forsberg, Hilary W. Heuer, Kejal Kantarci, Eliana Marisa Ramos, Howard J. Rosen, Bruce L. Miller, William W. Seeley, Suzee E. Lee, ARTFL/LEFFTDS/ALLFTD Consortia

**Affiliations:** ^1^ Memory and Aging Center, Department of Neurology, Weill Institute for Neurosciences, University of California, San Francisco, San Francisco, CA, USA; ^2^ Institute for Medical Imaging Technology, Ruijin Hospital, Shanghai Jiao Tong University School of Medicine, Shanghai, China; ^3^ Department of Neurology, Memory and Aging Center, University of California San Francisco, San Francisco, CA, USA; ^4^ Mayo Clinic, Rochester, MN, USA; ^5^ Memory and Aging Center, Weill Institute for Neurosciences, University of California, San Francisco, San Francisco, CA, USA; ^6^ David Geffen School of Medicine, University of California, Los Angeles, Los Angeles, CA, USA

## Abstract

**Background:**

Previous studies demonstrate that certain asymptomatic *MAPT* variant carriers (Asymp‐*MAPT*) exhibit altered baseline gray matter volume (GMV) and functional connectivity (FC) profiles. It remains unclear whether these imaging measures may herald risk for earlier conversion to the symptomatic phase. To address this, we stratified Asymp‐*MAPT* based on baseline GMV and longitudinal FC changes to assess whether any Asymp‐*MAPT* subgroups showed signs of longitudinal neurodegeneration or cognitive decline.

**Method:**

We studied 25 Asymp‐*MAPT* recruited from the UCSF Memory and Aging Center and ALLFTD Consortia. Using a median split, we divided Asymp‐*MAPT* into groups with smaller (LowGMV; *n* = 12) and larger baseline GMV (HighGMV; *n* = 13) within regions atrophied in symptomatic *MAPT* carriers (*n* = 20). Next, we stratified Asymp‐*MAPT* based on longitudinal FC changes using k‐means clustering on rates of change in FC for four intrinsic connectivity networks associated with common *MAPT*‐related syndromes: salience (SN), corticobasal syndrome (CBS), progressive supranuclear palsy (PSP), and default mode (DMN) networks. We compared Asymp‐*MAPT* subgroups from each stratification on baseline and longitudinal changes in demographics, plasma neurofilament light (NfL) concentration, GMV within 246 Brainnetome regions, and neuropsychological test scores.

**Result:**

Stratification of Asymp‐*MAPT* based on baseline GMV showed that LowGMV had higher education levels compared to HighGMV, with no other demographic differences. Though LowGMV and HighGMV did not differ in baseline NfL, LowGMV showed greater longitudinal NfL increases. These groups did not differ in baseline or longitudinal GMV changes. LowGMV had lower baseline attention and working memory scores, and greater longitudinal decline in set‐shifting and visuospatial performance. Stratification based on FC changes yielded one group (DecFC; *n* = 17) exhibiting longitudinal decreases in the SN, PSP, and DMN and another (IncFC; *n* = 8) showing increases in each network. DecFC and IncFC did not differ in demographics, baseline or longitudinal NfL, or baseline GMV. Longitudinally, DecFC showed greater decline in posterior superior temporal and inferior parietal cortices. DecFC showed lower baseline attention and semantic fluency scores, and greater longitudinal decline in set‐shifting and visuospatial memory performance.

**Conclusion:**

Lower baseline GMV within regions atrophied during the symptomatic phase and longitudinal FC declines within *MAPT*‐relevant networks may signal earlier symptomatic conversion in Asymp‐*MAPT*.

